# Methotrexate plus idarubicin improves outcome of patients with primary central nervous system lymphoma

**DOI:** 10.18632/oncotarget.15899

**Published:** 2017-03-04

**Authors:** Ni Fan, Lu Zhang, Xiaoping Xu, Bobin Chen, Chen Zhu, Pei Li, Zi Chen, Tianling Ding, Yan Ma, Yan Yuan, Zhiguang Lin

**Affiliations:** ^1^ Department of Hematology, Huashan Hospital, Fudan University, Shanghai, China

**Keywords:** PCNSL, methotrexate, idarubicin, PFS

## Abstract

Primary central nervous system lymphoma (PCNSL) is a rare form of non-Hodgkin lymphoma with poor long-term survival. This study assessed methotrexate (MTX) plus idarubicin (IDA) in treating patients of PCNSL comparing to MTX alone therapy. A total of 100 patients were retrospectively enrolled and subjected to MTX alone (*n* = 52) and MTX plus IDA (*n* = 48). The completed response (CR) rate in patients treated with MTX plus IDA was 62.50%, and overall response (OR) rate was 79.17%, which in MTX alone cohort were 42.31% and 63.46% respectively. Median progression-free survival (PFS) of patients treated with MTX plus IDA was significantly better than those treated with MTX alone (18.35 months *vs*.8.45months, *P* = 0.000). The MTX plus IDA regimen exhibited a significantly better control of PCNSL. Further studies would be needed to confirm these results.

## INTRODUCTION

Primary central nervous system lymphoma (PCNSL) is a rare extranodal form of non-Hodgkin lymphoma (NHL), which occurs intracerebrally or intraocularly, with poor long-term survival. These lymphomas exclusively arise in the central nervous system (CNS) such as the brain parenchyma, spinal cord, eyes and/or cranial nerves; and accounts for less than 1% of all NHLs and approximately 2-3% of all brain tumors.[1] Histologically, approximately 90% of CNS lymphomas are diffuse large B cell lymphoma (DLBCL); and the rest includes Burkitt’s, lymphoblastic and T-cell lymphomas.[2] In 2008, the WHO classification of tumors of hematopoietic and lymphoid tissues defined the majority as primary DLBCL of the CNS (CNS-DLBCL).[1] Human immunodeficiency virus (HIV) infection induces the risk of developing PCNSL for up to 3,600 folds compared to general populations. However, unlike immunocompetent patients, patients with HIV-associated PCNSL have unique clinical characteristics.[3]Thus, they were excluded from the category of PCNSLs. Molecularly, it has been well-established that all CNS-DLBCLs are positive for B-cell markers (CD20 and CD79a); and approximately 90% express MUM1 and 60-80% express BCL-6,[1] which indicate a unique activated B-cell-like immunophenotype.[4]

Clinically, although similar features exist between CNS and systematic DLBCLs, the prognosis of PCNSL is much worse than other extranodal DLBCLs, leading to different treatment options. Conventionally, methotrexate (MTX)-based chemotherapy has been a prerequisite treatment option in upfront regimens for PCNSL, while MTX combined with other medications may improve therapeutic outcomes such as cytarabine (Ara-C), idarubicin (IDA), and temozolomide. These medications have been selected based on their capability to penetrate the blood-brain barrier (BBB) and their efficacy against systemic lymphomas. However, the precise superiority of these polychemotherapies remains to be determined.[2,5–8] Therefore, in this study, we assessed MTX together with IDA as upfront regimens for managing newly diagnosed CNS lymphoma in patients.

## PATIENTS AND METHODS

### Patients

A total of 100 patients newly diagnosed with PCNSL between January 2007 and November 2016 in Huashan Hospital, the general department and the north branch, (Shanghai, China) were retrospectively enrolled in this study, with the enrollment criteria of HIV-negative and non-immunosuppression-related PCNSL. These patients were pathologically reconfirmed according to the 2008 WHO classification of lymphomas [1]. Clinicopathological data were collected, including clinical presentations, diagnoses, regimens, therapeutic outcomes, second-line treatments, toxicity reactions, and survival durations. Signed informed consent was obtained from all patients or guardians.

All patients underwent gadolinium-enhanced cranial magnetic resonance imaging (MRI) scan and/or positron emission tomography/computed tomography (PET/CT) before and after chemotherapy to evaluate the response to treatment. Serum lactate dehydrogenase (LDH), total creatinine clearance (CCr), B ultrasound of lymph nodes and abdomen, slit-lamp examination, as well as bone marrow biopsy and aspiration were also conducted in all patients.

### Treatment regimens and response assessment

Based on clinicopathological data, each patient was treated with MTX-alone (*n* = 52; median intravenous dose of MTX 3.2g/m^2^, range 10-8g/m^2^) or MTX plus IDA (*n* = 48; median intravenous dose of MTX 3.0g/m^2^, range 1.5-8g/m^2^, and IDA 8.5mg/m^2^, range 7-12g/m^2^). In the MTX/IDA group, MTX was used on day 1, IDA was used on day 2, and dexamethasone (DEX) of 15mg/d was used on day 1-3. In the MTX group, MTX was used on day 1 and DEX was in accordance with MTX/IDA group. Hyperhydration (2000-3000ml per day) and urine alkalinization were initiated before MTX infusion. Leucovorin rescue (20-25mg every 6 hours) was begun 24 hours after MTX infusion for at least 8 times until elimination. Treatments were repeated every 3 weeks except in case of progression and/or unacceptable toxicity or patient refusal.

Every patient was reassessed comprehensively after 4 courses of chemotherapy. Objective treatment responses were assessed according to the International Primary CNS Lymphoma Collaborative Group (IPCG) consensus guidelines, [9] which include: complete response (CR), partial response (PR), progressive disease (PD), and stable disease (SD). Patients who achieved CR continued to receive 2 more courses with the same regimens, followed with extended interval chemotherapies (every 2 to 3 months) for another 2 courses. Patients who presented with PR received another 2 courses as before and then received reassessment to confirm the treatment response. As CR achieved, the same protocol was followed as mentioned. Otherwise, a more aggressive regimen was administered with temozolomide added to the previous one. Patients who presented with SD or PD were switched to high-dose Ara-C chemotherapy. OR was referred as the sum of CR and PR. Response and side effects were evaluated after each course of chemotherapy, and acute toxicity was graded based on the WHO hematological toxicity scale.[10] The primary endpoint events were the progression of the lymphomas and the death of the patients. The secondary endpoint events were the CR achievement and the relapse after it. The last follow-up was conducted in November 30, 2016.

### Statistical analysis

Descriptive and explorative data analyses of all parameters were done. Quantitative parameters were tested for normal distribution. The Kruskal-Wallis and Mann-Whitney U tests were used for quantitative parameters; the χ^2^ square test and the Fisher's test for non-quantitative parameters. Survival and follow-up were calculated from the date of PCNSL diagnosis to the time of death, recurrence, progression, or last follow-up. Univariate analysis was performed using log-rank test, and survival distributions were analyzed by the Kaplan-Meier curve and log-rank test. Multivariate analysis was done by Cox regression model.

All tests were two-sided and *P* < 0.05 was considered to be statistically significant. All statistical analyses were performed with SPSS (Version 20.0; SPSS Inc) and Graph Pad Prism (Version 6.0).

## RESULTS

### Patient characteristics

The main patient characteristics are listed in Table I. Specifically, median age of patients was 56.5 years (range, 21-74 years) in the IDA/MTX group and 56 years in the MTX group (range, 35-74 years). The median Karnofsky performance scores (KPS) were 50 in the IDA/MTX group and 70 in the MTX group, both with the same range from 20 to 80. Fifteen patients had elevated serum LDH levels in both the IDA/MTX group (28.8%) and the MTX group (31.3%), respectively. Five patients (5/28) in the IDA/MTX group had lymphomatous cells in the CSF after cytology examination with six (6/26) in the MTX group. Furthermore, high CSF protein levels were identified in 22 patients (22/28) in the IDA/MTX group and 21 patients (21/26) in the MTX group. Besides, 33 patients (68.8%) in the IDA/MTX group and 31 patients (59.6%) in the MTX group had lymphoma that involved deep sites of the brain including the periventricular regions, basal ganglia, corpus callosum, brainstem, and/or cerebellum. Moreover, 31 patients (64.6%) in the IDA/MTX group had multiple lesions *vs*.27 patients (51.9%) in the MTX group.

**Table I:** Patient characteristics

Characteristics	IDA/MTX(*n*=48)	MTX(*n*=52)	*P* value^c^
**Age, years(median)**	21-74 (56.5)	35-74 (56)	0.423
**>60years, number**	18(37.5%)	13(25.0%)	0.177
**Sex**			0.342
**Male**	33(68.8%)	31(59.6%)	
**Female**	15(31.3%)	21(40.4%)	
**KPS on admission(median)**	20-80(50)	20-80(70)	0.344
**Diagnosis**			0.48
**Surgery**	31(64.6%)	30(57.7%)	
**Biopsy**	17(35.4%)	22(42.3%)	
**Pathology**			0.295
**B-cell lymphoma**	8(16.7%)	5(9.6%)	
**DLBCL**	40(83.3%)	47(90.4%)	
**Others**	0	0	
**Extension**			0.2
**Single mass lesion**	17(35.4%)	25(48.1%)	
**Multiple**	31(64.6%)	27(51.9%)	
**PET-CT SUV(median)**	3.3-35.4(15.6)	8.9-48.6(14.3)	0.93
**Total GFR, ml/min(median)**	48.48-132.88(89.8)	65.1-133.9(86.16)	0.567
**Elevated serum LDH level**^a^	15(28.8%)	15(31.3%)	0.794
**Elevated serum β2-M**^a^	13/46(28.3%)	13/47(27.7%)	0.948
**Elevated urine β2-M**^a^	14/35(40%)	13/32(40.6%)	0.958
**Positive CSF cytology examination**^a^	5/28(17.9%)	6/26(23.1%)	0.634
**Elevated CSF pressure**^a^	12/28(42.9%)	5/26(19.2%)	0.062
**Elevated CSF WBC count**^a^	8/28(28.6%)	6/26(23.1%)	0.645
**Elevated CSF proteinlevel**^a^	22/28(78.6%)	21/26(80.8%)	0.841
**BMI, kg/m2(median)**	15.67-33.66(22.9)	17.9-31.14(23.18)	0.282
**Involvement of deep structures**^b^	33(68.8%)	31(59.6%)	0.907
**Front lobe**	22	21	
**Parietal lobe**	10	11	
**Temporal lobe**	14	10	
**Occipital lobe**	10	4	
**Basal ganglia**	11	12	
**Brain stem**	4	4	
**Cerebellum**	11	8	
**Periventricular regions**	14	7	
**Spinal cord**	0	1	
**callosum**	8	8	
**thalamus**	4	7	
**eyes**	4	6	
**Clinical Manifestation**			
**intracranial hypertension**	28	24	
**movement disorders**	24	20	
**sensory dysfunction**	2	8	
**speech disorders**	12	10	
**visual disturbance**	8	10	
**cognitive disorders**	13	18	
**facioplegia**	5	3	
**convulsion**	5	3	

Abbreviations: KPS, Karnofsky performance score; DLBCL, diffuse large B-cell lymphoma; PET-CT, positron emission tomography/computed tomography; SUV, standard uptake value; GFR, glomerular filtration rate;β2-M,β2-microglobulin; LDH, serum lactate dehydrogenase; CSF, cerebrospinal fluid; WBC, white blood cell; BMI, body mass index.

Normal ranges: LDH, 125-225U/L; serumβ2-M, 0.70-1.80mg/l; urineβ2-M, 0-0.25mg/l; CSF pressure, 80-180cmH2O; CSF WBC count, 0-8*10^6^/L; CSF protein level, 150-450 mg/L.

^a^The number of patients with high levels *vs*. the total number of assessed patients.

^b^Tumor involved in deep structures of the brain including periventricular regions, basal ganglia, corpus callosum, brainstem, and/or cerebellum.

^c^Quantitative parameters were tested by the Mann-Whitney U tests and non-quantitative parameters were tested by the χ2 square test and Fisher's test.

Descriptive and explorative data analyses of all parameters were done. Quantitative parameters were tested for normal distribution. To test the differences of patient baseline characteristics between the two groups, the Mann-Whitney U tests were used for quantitative parameters, with the χ2 square test and the Fisher's test for non-quantitative parameters. Data shows that there are no significant differences in the characteristics of the two groups.

### Treatment responses

These patients were followed up for a median duration of 18.33months (range 1.4-105.27 months) and the last follow-up was completed on November 30, 2016. In Table II, complete remission (CR) rate was identified in 62.50% of patients in the IDA/MTX cohort and in 42.31% of patients who received MTX monotherapy (*P* = 0.043). Overall response (OR) rate was 79.17% in the IDA/MTX group (30 CR, 8 PR) and 63.46% in the MTX group (22CR, 11PR).

**Table II:** Outcomes of the two treatment options

	IDA/MTX(*n* = 48)	MTX(*n* = 52)	*P* valuea
**CR rate,%**	62.50%	42.31%	***0.043***
**OR rate,%**	79.17%	63.46%	0.084
**PFS, month (median)**	0.9-69.77 (18.35)	1.27-32 (8.45)	***0.000***
**OS, month (median)**	1.57-105.27(22.93)	1.40-41.47(16.38)	***0.013***

Abbreviations: CR, complete response; OR, overall response, includes CR and PR; PFS, progression-free survival; OS, overall survival.

CR rate and OR rate were tested by the χ2 square test. PFS and OS were tested by log-rank test.

**Figure 1:**
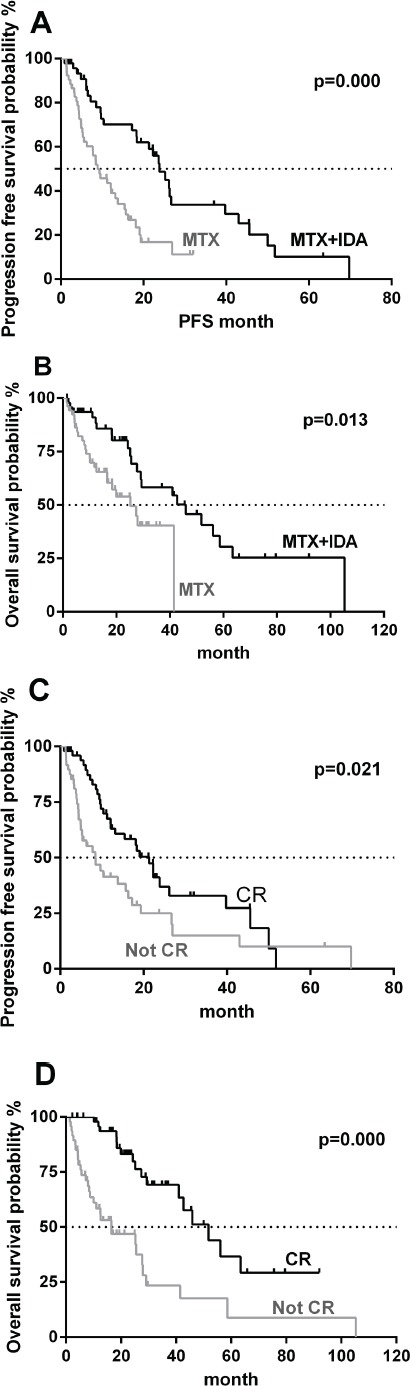
Kaplan-Meier curve analysis of progression-free survival (PFS) and overall survival (OS) **A**. PFS between MTX-alone and MTX plus IDA. **B**. OS between MTX-alone and MTX plus IDA. **C**. PFS stratified by achieving completed response (CR) or not. **D.** OS stratified by achieving CR or not.

Median progression-free survival (PFS) in these two treatment options was 18.35months and 8.45 months, respectively, and the difference was statistically significant (*P* = 0.000, Figure 1A). Median OS was 22.93 months in the IDA/MTX group and 16.38 months in the MTX group, the difference of which was also statistically significant (*P* = 0.013, Figure 1B).

### Univariate analysis

Univariate analyses were done for the factors that may affect PFS, and results were showed in Table III.

**Table III:** Results of univariate analyses

Factors	PFS (median, month)	χ2	*P* value^a^
**age**		0.234	0.630
**>60 years**	6.50		
**≤60 years**	11.03		
**KPS**		0.154	0.696
**≥70**	9.59		
**<70**	10.33		
**CR or not**		4.438	0.038
**yes**	14.32		
**no**	5.45		
**diagnosis**		0.811	0.370
**surgery**	8.47		
**biopsy**	15.43		
**LDH**		0.180	0.673
**high**	10.38		
**normal**	9.15		
**urine β**_2_**-M**		0.007	0.932
**high**	10.43		
**normal**	8.75		
**serum β**_2_**-M**		0.344	0.559
**high**	8.12		
**normal**	10.43		
**CSF pressure**		0.466	0.498
**high**	8.57		
**normal**	11.03		
**CSF protein**		0.323	0.572
**high**	11.03		
**normal**	6.5		
**CSF WBC**		0.013	0.908
**high**	11.55		
**normal**	9.02		
**Involvement of deep structures**		0.015	0.904
**yes**	9.65		
**no**	9.95		
**Extension**		0.002	0.964
**single**	9.07		
**multiple**	10.02		

Abbreviations: KPS, Karnofsky performance score; CR, complete response; β2-M, β2-microglobulin; LDH, lactate dehydrogenase; CSF, cerebrospinal fluid; WBC, white blood cell.

^a^Tested by log-rank test.

Results showed that PFS in all patients who had achieved CR (both groups combined) was significantly longer than those who did not. In patients who did achieve CR, median PFS was 14.32 months (range, 0.9-51.77)vs. only 5.45 months (range,1.27-69.77) in patients who did not achieve CR (*P* = 0.038, Figure 1C).

Median PFS was 11.03 months in patients younger than 60 years (69 cases) and 6.5 months in those>60 years of age (31cases), although this difference did not reach statistical significance (*P* = 0.630). And also, the comparison between patients with higher KPS (48 cases, ≥70) and those < 70 (52 cases) was not statistically different either (*P* = 0.0.696).

### Multivariate analysis

Achieving CR, therapeutic regimen and patients’ age were concluded into the Cox regression model for multivariate analysis, and results were showed in Table IV. Therapeutic regimen (*P* = 0.000) and achieving CR or not (*P* = 0.021) were independent prognostic factors for PFS, while age was excluded.

**Table IV:** Results of multivariate analysis

	HR	95.0% CI		Sig.
		Lower	Upper	
Age	0.997	0.971	1.023	0.794
Therapeutic regimen	2.876	1.64	5.042	***0.000***
CR or not	1.811	1.095	2.993	***0.021***

Abbreviations: HR, hazard ratio; 95.0%CI, 95.0% confidential interval; Sig. significance value P.

### Toxicity of chemotherapy

Data on chemotherapy toxicity are shown in Table V. In brief, 13 patients (27.1%) in the IDA/MTX group and 18 patients (34.6%) in the MTX group had Grade 3/4 hematological toxicity. 10 patients (20.8%) in the IDA/MTX group and 12 patients (23.1%) in the monotherapy group developed fever during neutropenic episodes, with totally 20 patients had pneumonia. With regard to other adverse events, nausea (grade 2) was observed in 7 patients in the IDA/MTX group and 8 patients in the MTX group. Liver dysfunction (grade 3) was observed in one patient in the IDA/MTX group. Three patient also in the IDA/MTX group developed deep venous thrombosis. Renal dysfunction (Grade 2) was observed in 6 patients in the IDA/MTX group and 5 patients in the MTX group. High fever (grade 3 or more) and cardiotoxicity was not observed in either group.

**Table V:** Chemotherapy toxicity using the National Cancer institute Common Toxicity Criteria

	IDA/MTX (*n*=48)	MTX(*n*=52)	*P* value^a^
**Hematologic toxicities**			
**Neutropenia G3-4**	9 (18.75%)	11 (21.15%)	0.764
**Thrombocytopenia G3-4**	3 (6.25%)	5 (9.62%)	0.717
**Anemia G3-4**	1 (2.08%)	2 (3.85%)	1
**Non-hematologic toxicity**			
**Nausea G2**	7 (14.58%)	8 (15.38%)	0.911
**Liver dysfunction G3**	1 (2.08%)	4 (7.69%)	0.364
**DVT G2**	3 (6.25%)	0 (0%)	0.107
**Renal dysfunction G2**	6 (12.5%)	5 (9.62%)	0.645
**Drug eruption G2**	4 (8.33%)	8 (15.38%)	0.362
**Febrile neutropenia G2**	10 (20.83%)	12 (23.08%)	0.787
**Numbness G2**	2 (4.17%)	1 (1.92%)	0.606

Abbreviations: DVT, deep venous thrombosis.

^a^ Comparisons between the two groups were tested by the χ2 square test and Fisher's test.

## DISCUSSION

Up to now, PCNSL accounts for approximately 2.2% of all primary tumors in the central nervous system.[1, 11, 12] However, its incidence has increased since 2000, particularly in the elderly, [11–13] accounting for more than half of PCNSL patients.[14] After treatment, these patients have also shown the highest risk of therapeutic neurotoxicity. To date, treatment outcome of PCNSL, like most other cancers, has continued to progress over the past decades. Nevertheless, no consensus therapy has been established at present, leading to poor long-term survival.[2, 15–17] Furthermore, current chemotherapies have demonstrated high rates of late neural complications in elderly patients who are ineligible for aggressive chemotherapies.[18] For example, a previous study revealed that after intensive regimens of chemotherapy were administered, approximately 90% of patients older than 60 years of age developed delayed neurotoxicity and died from related complications rather than lymphoma *per se*.[8] Evidently, for these patients, moderate chemotherapy alone plus other chemotherapeutic agents might be a reasonable option. Thus, it is crucial to develop such novel strategies. In the current study, we assessed the clinical outcome of patients after receiving MTX combination therapy. We found that 52 immunocompetent patients with PCNSL had a median PFS of 8.45 months by receiving MTX monotherapy, whereas MTX plus IDA treatment revealed a significantly improved PFS of 18.35 months in 48 patients.

In many previous studies, other clinical factors have been shown to influence PCNSL therapeutic outcomes such as age, KPS, serum LDH level, CSF protein level, and tumors that occur in deep cerebral sites [19–21]. Thus, age and KPS have been widely used to predict the outcome of patients. Indeed, other recent studies have demonstrated that age over 60 year old and/or KPS < 70 predicted poor survival of patients.[8,20–23] Furthermore, high serum LDH levels have been found to be significantly associated with low treatment response rate, and used as an independent prognostic predictor for PCNSL.[19, 24, 25] Moreover, Levinson *et al*. used 45 mg/dl and 60 mg/dl as cut-off points for CSF protein levels in patients younger than 60 years old and elderly patients, respectively; [19] which could serve as an indicator for tumor behaviors, [19, 20] allow a more aggressive clinical course, and help to determine the lymphoma involved in the deep sites of the brain.[19] Besides factors mentioned above, we also compared ways of diagnosis, tumor numbers (single or multiple), PET-CT SUV, total GFR levels, elevated levels of serum/urine β2-M, elevated CSF pressure and CSF WBC counts, as well as involvement of deep structures or not between the two groups. By univariate analysis and multivariate analysis, results showed no statistical discrepancy, indicating that these contributing factors could be ignored in terms of PFS. In addition, we found that PFS of patients who did achieve CR was significantly longer than patients who did not achieve CR (*P* = 0.021), in consistent with some reports that the CR rate is significantly associated with long-term survival.[26, 27]

Chemotherapy is the basic and standard approach to control PCNSL. However, the BBB has been a significant obstacle for the clinical administration of effective cytotoxic agents to eliminate encephalic lesions. Most medications, which would otherwise be capable of treating CNS disorders, may be inaccessible to these very regions for their efficacy. Recent studies of treatments for PCNSL were listed in Table VI. Comparing with these studies, the current study have showed equivalent or superior efficacy in treating PCNSL, whether in the response rate or PFS. And also, there are some problems in part of these studies, such as poor repeatability, strong toxic effects, high cost and shortage of sources, while IDA has been widely used in the treatment of leukemia and lymphoma. IDA is an analog of daunorubicin and a semi-synthetic antineoplastic anthracycline characterized by high lipophilicity, could result in increased rates of cellular uptake compared with other anthracyclines; [28, 29] suggesting that increased lipophilicity could make IDA an important candidate for the treatment of PCNSL. Previous studies have exhibited the efficacy of IDA-containing regimens on PCNSL, although one study failed to reveal its significant effect on patients without MTX [30] and other studies demonstrated promising results using IDA-containing polychemotherapies based on a high-dose of MTX.[31–34] However, it was also recognized that high responses were associated with acute toxicity; thus, improvement of the efficacy-toxicity balance is crucial in the treatment of these patients. The MTX and IDA regimen has been rarely employed to treat PCNSL in literatures and in our current study; thus, we assessed their therapeutic efficacy with acceptable hematological toxicity. Our current data revealed that the combination of MTX with IDA was in fact effective for treating PCNSL. Since there is currently no consensus on the guidelines for the dosage of IDA in treating PCNSL, Olivier G *et al*. recommended a 16 mg/m^2^ dose of IDA administrated together with MTX.[31] Unfortunately, this recommendation was not supported by other clinical trials.[30, 35, 36] Thus, in the current study, we used a median IDA dose of 8.5mg/m^2^. Further studies are needed to establish the standard dosage for IDA.

**Table VI:** Recent studies of treatments for PCNSL

Refs.	Patients No.	Median age(range)	treatment	OR% (CR %)	Median OS (months)	Median PFS (months)	Year
K.INA.LY *et al*.[45]	12	62.5 (19-78)	M+R	91 (58)	_	22	2016
Liren Qian et al.[46]	19	54 (24-75)	R+IDA+D+A+M+ IT(R+M+D+A)	89 (94)	_	_	2016
Mocikova.H *et al*.[47]	49	63 (31-70)	R+M+V+ PCZ±WBRT/A	65 (41)	28.6	22.9	2016
Kansara R *et al*.[48]	29	61 (18-80)	R+M± WBRT	50 (37)	_	_	2015
Omuro A *et al*.[49]	32	57 (23-67)	R+M+PCZ+V	44 (94)	_	_	2015
Liu J *et al*.[50]	18	51 (34-83)	R+M+A+D	94.5 (55.6)	22	_	2015
Ichikawa T *et al*.[51]	24	64.6(mean) (50-78)	M+C+DXR+V+P± WBRT	100 (87.5)	33	13	2014
Wang XX *et al*.[52]	21	52 (34-76)	M+A	61.9 (38.1)	_	_	2014
	20	53.5 (18-72)	M+T	70 (45)	_	_	2014
Ghesquieres *et al*.[53]	36	66 (61-69)	C+V+P M+C+V+P M+A	61 (53)	16	16	2010

Abbreviations: M, methotrexate; R, rituximab; V, vincristine; PCZ, procarbazine; IDA, idarubicin; A, cytosine arabinoside; D, dexamethasone; IT, intrathecal; C, cyclophosphamide; DXR, doxorubicin; P, prednisone; T, temozolomide; WBRT, whole brain radiotherapy; L, lomustine.

Furthermore, MTX-based chemotherapy has been a prerequisite therapy in upfront regimens for PCNSL, and it has been recommended by National Comprehensive Cancer Network that the preferred dosage of MTX was 3.5-8.0 g/m^2^.[37] Delayed neurotoxicity commonly associated with leukoencephalopathy is one of the most serious complications in MTX treatment for PCNSL.[38–41] However, since the absence of related multi-center prospective studies, the efficacy of MTX with a dose < 3.5g/m^2^ combined with other chemotherapeutics was still unknown. The relatively smaller dosage of MTX-containing regimen with/without radiotherapy was comparable to intensive chemotherapies (Table VII). For example, Yamanaka R *et al*. revealed that a modest dose of MTX (500 mg/m^2^) followed by reduced-dose WBRT (20-30 Gy) was a feasible approach with minimized serious toxicity.[42] Other previous studies demonstrated that a combination chemotherapy containing 1 g/m^2^ of MTX without radiotherapy also achieved comparable outcomes in elderly patients who were susceptible to neural complications.[43, 44] In the current study, our treatment protocol that contained an intravenous average MTX dose of 3.0g/m^2^ produced similar therapeutic outcomes, suggesting that a regimen containing a relatively lower dose of MTX could also fit patients with PCNSL, at least in Chinese patients.

**Table VII:** Studies of relatively lower dosage of MTX-containing regimen

Study	No. of patients	Median age(year)	Initial treatment	CR rate%	PFS, median (month)
DeAngelis LM. et al.[54]	98	56.5	MTX2.5g/m^2^+VCR+PCZ+IT+Ara-C	58	24.0
Yamanaka R. *et al*.[42]	32	61.3	MTX 500 mg/m^2^ +CTX +THP +VP16 +VCR +PCZ +Pred	100	35
Taoka K. *et al*.[43]	17	67	MTX 1 g/m^2^ +IT +MCNU +PCZ +mPSL	41	20.0
Freilich R.J *etal*.[55]	13	74	MTX 1-3.5 g/m^2^ +IT +PCZ ±VCR/TTP/Ara-C	76.9	-
Jalaeikhoo H *et al*.[56]	51	50.3	MTX2.5g/m^2^+VCR+Ara-C	_	32 (median OS)

Abbreviations:PFS, Progression-free free survival; MTX, methotrexate; IT, intrathecal (administration); PCZ,procarbazine; VCR, vincristine; TTP, thiotepa; Ara-C, cytarabine; CTX,cyclophosphamide;VP-16, etoposide; THP,pirarubicin;MCNU, ranimustine; mPSL, methylprednisolone; Pred,prednisone.

In addition, WBRT is a standard clinical approach for treating PCNSL, with a CR rate of 80-90%.[2, 57] However, even with a dose of up to 60 Gy, the prognosis of patients remained poor, because majority of patients had a relapse disease after WBRT.[57] Moreover, acute encephalopathy and delayed neurotoxicity could also occur in most patients.[38, 58, 59] Thus, WBRT is recommended to be reserved for patients who fail to achieve a CR or show disease progression after chemotherapy.[2] In order to avoid the impact of radiotherapy on the outcomes of patients, those who had received radiation treatments were excluded in the current study.

Autologous stem cell transplantation (HDT-ASCT), which has been introduced into the strategy, has been reported to be effective in managing primary, relapsed, or refractory PCNSL [60–63]. However, it has not been employed as a standard strategy by far. The superiority of HDT-ASCT compared with the conventional chemotherapy regimens is still under investigation [64, 65]. Moreover, HDT-ASCT is more aggressive than the regimens, and the cost is a heavy burden for the patients.

OS, which was evaluated as well in our study, showed a median value of 22.93 months in the IDA/MTX group, longer than 16.38 months in the MTX group, with statistically difference (*P* = 0.013). However, it was thought to be relatively less reliable to assess the therapeutic effect of these two treatments than response rate and PFS, since the patients had received different therapeutic schemes after progression, like cytarabine and temozolomide. Definitely, it was not prudent to make a conclusion that OS has been improved after all these variable regimens.

Our study provided a promising option for the patients with PCNSL; nevertheless, our research has intrinsic limitations in consideration that it is a retrospective, single-institution analysis. The matching of the two patient cohorts are suboptimal by reason that patients were not prospectively assigned to the different treatment groups using prognostic factors for randomization. Thus, future well-controlled prospective studies are required to confirm the current data.
